# Supplementary material for: The association between educational level and multimorbidity among adults in Southeast Asia: A systematic review
Source: PLoS One. 2021 Dec 20;16(12):e0261584. doi: 10.1371/journal.pone.0261584 (PMC8687566; doi:10.1371/journal.pone.0261584)
Supplement: S1 Table — (DOCX) [file pone.0261584.s001.docx]

**S1 Table Search query of databases.**

| **Database** | **Search query** | **Results** |
| --- | --- | --- |
| Scopus | TITLE-ABS-KEY ( "multi-morbidity" OR "multimorbidity" OR "multi-morbidities" OR "multimorbidities" OR "multimorbid" OR "multiple morbidities" OR "multiple morbidity" OR "multiple conditions" OR "multiple diseases" OR "multiple chronic diseases" OR "multiple chronic conditions" OR "multiple illnesses" OR "multiple diagnoses" OR "multi-pathology" OR "co-morbid" OR comorbidity OR "co-morbidity" OR "co-morbidities" OR "comorbidities" ) AND ALL ( education OR literacy OR "educational status" OR "educational level" OR "educational attainment" ) AND ALL ( "Southeast Asia" OR "Association of Southeast Asian Nation" OR "ASEAN" OR brunei OR cambodia OR "East Timor" OR indonesia OR laos OR malaysia OR myanmar OR philippines OR singapore OR thailand OR vietnam ) AND ( LIMIT-TO ( LANGUAGE , "English" ) ) | 3955 |
| *PubMed* | (("multi-morbidity"[Title/Abstract] OR "multimorbidity"[Title/Abstract] OR "multi-morbidities"[Title/Abstract] OR "multimorbidities"[Title/Abstract] OR "multimorbid"[Title/Abstract] OR "multiple morbidities"[Title/Abstract] OR "multiple morbidity"[Title/Abstract] OR "multiple conditions"[Title/Abstract] OR "multiple diseases"[Title/Abstract] OR "multiple chronic diseases"[Title/Abstract] OR "multiple chronic conditions"[Title/Abstract] OR "multiple illnesses"[Title/Abstract] OR "multiple diagnoses"[Title/Abstract] OR "multi-pathology"[Title/Abstract] OR "co-morbid"[Title/Abstract] OR comorbidity OR "co-morbidity"[Title/Abstract] OR "co-morbidities"[Title/Abstract] OR "comorbidities"[Title/Abstract]) AND (education OR literacy OR "educational status" OR "educational level" OR "educational attainment")) AND ("Southeast Asia" OR "Association of Southeast Asian Nation" OR "ASEAN" OR brunei OR cambodia OR "East Timor" OR indonesia OR laos OR malaysia OR myanmar OR philippines OR singapore OR thailand OR vietnam) Filters: English, from 1990/1/1 - 2021/6/15 | 591 |
| *ProQuest* | ab("multi-morbidity" OR "multimorbidity" OR "multi-morbidities" OR "multimorbidities" OR "multimorbid" OR "multiple morbidities" OR "multiple morbidity" OR "multiple conditions" OR "multiple diseases" OR "multiple chronic diseases" OR "multiple chronic conditions" OR "multiple illnesses" OR "multiple diagnoses" OR "multi-pathology" OR "co-morbid" OR comorbidity OR "co-morbidity" OR "co-morbidities" OR "comorbidities") AND (education OR literacy OR "educational status" OR "educational level" OR "educational attainment") AND ("Southeast Asia" OR "Association of Southeast Asian Nation" OR "ASEAN" OR Brunei OR Cambodia OR "East Timor" OR Indonesia OR Laos OR Malaysia OR Myanmar OR Philippines OR Singapore OR Thailand OR Vietnam)  Limitation: 1990-01-01 - 2021-06-15; English | 3012 |

| **Exposure** | **Outcome** | **Location** |
| --- | --- | --- |
| education,  literacy,  educational status,  educational level,  educational attainment | multi-morbidity,  multimorbidity,  multimorbid,  multiple morbidities,  multiple morbidity,  multiple conditions,  multiple diseases,  multiple chronic diseases, multiple chronic conditions,  multiple illnesses,  multiple diagnoses,  multi-pathology | Southeast Asia,  Association of Southeast Asian Nation,  ASEAN,  Brunei,  Cambodia,  East Timor,  Indonesia,  Laos,  Malaysia,  Myanmar,  Philippines,  Singapore,  Thailand,  Vietnam |
