# Supplementary material for: The association between educational level and multimorbidity among adults in Southeast Asia: A systematic review
Source: PLoS One. 2021 Dec 20;16(12):e0261584. doi: 10.1371/journal.pone.0261584 (PMC8687566; doi:10.1371/journal.pone.0261584)
Supplement: S2 Table — (DOCX) [file pone.0261584.s002.docx]

**S2 Table NOS Checklist for selected studies (cross-sectional study).**

| Study  (First author (year))  **Cross-sectional**  (Total ten scores) | **Selection** | | | | | | | | | | | | | **Comparability** | | | **Outcome** | | | | | | | **Total scores for all** | **Level** |
| --- | --- | --- | --- | --- | --- | --- | --- | --- | --- | --- | --- | --- | --- | --- | --- | --- | --- | --- | --- | --- | --- | --- | --- | --- | --- |
|  | Representativeness | | | | Sample size | | Non-respondents | | | Ascertainment  (risk factor) | | | Total scores | On the basis of the study design or analysis and the control of confounders | | Total scores | Assessment | | | | statistical test | | Total scores |  |  |
|  | Truly representative  (1) | Somewhat representative  (1) | Selected group  (0) | No description  (0) | Justified  (1) | Not justified  (0) | Satisfactory  (1) | Unsatisfactory  (0) | No description  (0) | Validated  (1) | Non-validated  (0) | No description  (0) |  | Most important factor  (1) | Any additional factor  (1) |  | Independent blind assessment  (1) | Record linkage  (1) | Self-report  (0) | No description  (0) | clearly described & appropriate  (1) | not appropriate or described  (0) |  |  |  |
| Abdin (2020) [23] | 1 |  |  |  | 1 |  |  | 0 |  | 1 |  |  | 3 |  | 1 | 1 |  | 1 | 0 |  | 1 |  | 2 | 6 | Fair |
| Afshar (2015) [16] | 1 |  |  |  | 1 |  | 1 |  |  | 1 |  |  | 4 | 1 |  | 1 |  |  | 0 |  | 1 |  | 1 | 6 | Fair |
| Anindya (2020) [24] | 1 |  |  |  | 1 |  | 1 |  |  | 1 |  |  | 4 |  | 1 | 1 |  | 1 | 0 |  | 1 |  | 2 | 8 | Good |
| Aye (2019) [25] | 1 |  |  |  | 1 |  | 1 |  |  | 1 |  |  | 4 |  | 1 | 1 |  |  | 0 |  | 1 |  | 1 | 6 | Fair |
| Ba (2019) [26] | 1 |  |  |  | 1 |  | 1 |  |  | 1 |  |  | 4 |  | 1 | 1 |  |  | 0 |  | 1 |  | 1 | 6 | Fair |
| Chong (2012) [27] | 1 |  |  |  | 1 |  | 1 |  |  | 1 |  |  | 4 |  |  | 0 |  | 1 | 0 |  | 1 |  | 2 | 6 | Poor |
| Ha (2015) [15] | 1 |  |  |  | 1 |  | 1 |  |  | 1 |  |  | 4 |  | 1 | 1 |  | 1 |  |  | 1 |  | 2 | 7 | Good |
| Hussain (2015) [28] | 1 |  |  |  | 1 |  | 1 |  |  | 1 |  |  | 4 |  | 1 | 1 |  | 1 | 0 |  | 1 |  | 2 | 7 | Good |
| Liew (2011) [30] |  | 1 |  |  | 1 |  | 1 |  |  | 1 |  |  | 4 | 1 | 1 | 2 |  | 1 |  |  | 1 |  | 2 | 8 | Good |
| Marthias (2021) [31] |  | 1 |  |  | 1 |  | 1 |  |  | 1 |  |  | 4 |  | 1 | 1 |  | 1 | 0 |  | 1 |  | 2 | 7 | Good |
| Mwangi (2019) [32] | 1 |  |  |  | 1 |  |  | 0 |  | 1 |  |  | 3 |  |  | 0 |  |  | 0 |  | 1 |  | 1 | 4 | Poor |
| Pengpid (2017) [14] |  | 1 |  |  | 1 |  |  | 0 |  | 1 |  |  | 3 |  | 1 | 1 |  | 1 |  |  | 1 |  | 2 | 6 | Fair |
| Pengpid (2021) [33] |  | 1 |  |  |  | 0 | 1 |  |  | 1 |  |  | 3 |  | 1 | 1 |  |  | 0 |  | 1 |  | 1 | 5 | Fair |
| Picco (2016) [34] | 1 |  |  |  | 1 |  | 1 |  |  | 1 |  |  | 4 |  | 1 | 1 |  |  | 0 |  | 1 |  | 1 | 6 | Fair |
| Subramaniam (2014) [35] | 1 |  |  |  | 1 |  | 1 |  |  | 1 |  |  | 4 |  |  | 0 |  |  | 0 |  | 1 |  | 1 | 5 | Poor |
| Subramaniam (2017) [36] | 1 |  |  |  | 1 |  | 1 |  |  | 1 |  |  | 4 | 1 | 1 | 2 |  | 1 | 0 |  | 1 |  | 2 | 8 | Good |
| Tiptaradol (2012) [37] |  | 1 |  |  | 1 |  |  | 0 |  | 1 |  |  | 3 |  | 1 | 1 |  | 1 |  |  | 1 |  | 2 | 6 | Fair |

**S2 Table NOS Checklist for selected studies (cohort study).**

| Study  (First author (year))  **Longitudinal**  (Total nine scores) | **Selection** | | | | | | | | | | | | | | **Comparability** | | | **Outcome** | | | | | | | | | | | **Total scores for all** | **Level** |
| --- | --- | --- | --- | --- | --- | --- | --- | --- | --- | --- | --- | --- | --- | --- | --- | --- | --- | --- | --- | --- | --- | --- | --- | --- | --- | --- | --- | --- | --- | --- |
|  | Representativeness | | | | Non exposed cohort | | | Ascertainment | | | | Demonstration | | Total scores | On the basis of the design or analysis | | Total scores | Assessment | | | | Long enough follow-up | | Adequacy | | | | Total  scores |  |  |
|  | Truly representative  (1) | Somewhat representative  (1) | selected group  (0) | No description  (0) | Same community  (1) | Different source  (0) | No description  (0) | Secure record  (1) | structured interview  (1) | Written self -report  (0) | No description  (0) | Yes (1) | No (0) |  | Most important factor  (1) | Any additional factor  (1) |  | independent blind  (1) | Record linkage  (1) | Self-report  (0) | No description  (0) | Yes  (1) | No  (0) | complete follow up  (1) | subjects lost to follow up  (1) | No description of those lost  (0) | no statement  (0) |  |  |  |
| Hussin (2020) [29] | 1 |  |  |  | 1 |  |  |  |  | 0 |  | 1 |  | 3 |  | 1 | 1 |  |  | 0 |  |  | 0 | 1 |  |  |  | 1 | 5 | Fair |
