# Supplementary material for: The association between educational level and multimorbidity among adults in Southeast Asia: A systematic review
Source: PLoS One. 2021 Dec 20;16(12):e0261584. doi: 10.1371/journal.pone.0261584 (PMC8687566; doi:10.1371/journal.pone.0261584)
Supplement: S1 File — (PDF) [file pone.0261584.s004.pdf]

To enable PROSPERO to focus on COVID-19 submissions, this registration record has undergone basic automated checks for eligibility and is published exactly as submitted.
PROSPERO has never provided peer review, and usual checking by the PROSPERO team does not endorse content. Therefore, automatically published records should be treated as any other PROSPERO registration. Further detail is provided here.

Citation

Xiyu Feng, Matthew Kelly, Haribondhu Sarma. The association between educational level and multi-morbidity of non-communicable diseases (NCDs) among adults in Southeast Asia: Systematic Review. PROSPERO 2021 CRD42021259311 Available from:
https://www.crd.york.ac.uk/prospero/display_record.php?ID=CRD42021259311

Review question

What are the joint prospective associations between educational level with the outcomes of the prevalence of multi-morbidity of non-communicable diseases among adults in Southeast Asia?

Searches

I plan to use the five databases, namely, PubMed, ANU library, Scopus and Cochrane Library and Web of Science. No restriction of publication date but the literature must be English.

Types of study to be included

Cross-sectional, cohort and longitudinal study.

Condition or domain being studied

Multi-morbidity of non-communicable disease is defined as a person suffering from two or more chronic non-communicable diseases at the same time. Although the prevalence of multi-morbidity is relatively low in developing countries such as Thailand, ranging from 14% to 68%, compared to developed countries (14% to 90%), it is due to the substantial decline in mortality in childhood and childbirth and mortality of infectious diseases in these countries and the continued increase in the average life expectancy of the population. However, as aging is increasing in Southeast Asia, multi-morbidities and co-morbidities are becoming more common.

Participants/population

The study population of this topic will focus on the studies done on the adults in Southeast Asia (including Brunei, Philippines, Singapore, Vietnam, Laos, Cambodia, Thailand, Myanmar, Malaysia, Timor-Leste).

Intervention(s), exposure(s)

The level of education, as one of the factors of socioeconomic status (SES), is very important for the health of the population. This is because contemporary society is an information and a knowledge economy society. A higher level of education means that people will have more chances to find good jobs, better living environment, better eating habits, more attention to their health and thus health level will increase. Therefore, the purpose of taking the level of education above secondary school as exposure is to investigate whether higher education level will reduce the prevalence of multi-morbidity/comorbidity of non-communicable diseases among Southeast Asia.

Comparator(s)/control

The reason why illiterate or below secondary school level people are used as a control is because the living environment of this group of people is generally not as friendly as those with higher education and they are also more prone to various diseases and have a higher chance of suffering from multi-morbidity/comorbidity of non-communicable diseases.

Main outcome(s)

Multi-morbidity and comorbidity of non-communicable disease.

Additional outcome(s)

None.

Data extraction (selection and coding)

As for the assessing of the literature, we should use the JBI Critical Appraisal Tools to check the trustworthiness of literature which will be in the systematic review.This data and information can be listed in the table. The table of each article can contain the following messages such as (1) Author, Year, (2) Country, (3) Study type, (4) Study Population, (5) Male %/Age (years), (6) Definition of Multi-morbidity/Comorbidity of non-communicable diseases/NCDs, (7) Ascertainment of morbidities, and (8) results. Then, the data extraction results should be submitted to a third-party review.

Risk of bias (quality) assessment

The potential limitations are mainly reflected in the following aspects. The first is that some database access is difficult, which leads to the inability to retrieve some articles, reducing the validity of the outcomes. Secondly, there is a lack of literature published in related fields, making it hard to extract valid data for evaluation and analysis. Thirdly, the heterogeneity of the studies included is also a limitation since multi-morbidity or comorbidity of non-communicable disease is defined and measured in different ways in different studies. These limitations may lead to a decrease in the reliability and validity of the systematic review. Thus, in practice, these limitations should be avoided as much as possible.

Strategy for data synthesis

The qualitative study should be used narrative synthesis, which included the definition of multi-morbidity and comorbidity of non-communicable disease. And the quantitative analysis should be used on prevalence of the multi-morbidity and comorbidity of non communicable disease and the association between educational level and multi-morbidity/comorbidity among adults. If the sources of data found in the literature are similar, e.g., questionnaires or clinical data, then quantitative analysis can use meta-analysis. However, if the sources of data in the literature are very different, meta-analysis may not be used.

Analysis of subgroups or subsets

Qualitative study: Because different articles have different definitions of non-communicable disease comorbidity and multi-morbidity, and these definitions are narrative, this section uses narrative narrative synthesis to summarize the definition of non communicable disease multi-morbidity and comorbidity.

Quantitative analysis: As for the prevalence of the multi-morbidity and comorbidity of non-communicable disease and the association between educational level and non-communicable disease multi-morbidity/comorbidity among adults, it should be synthesized by cross-sectional, cohort and longitudinal studies.if the sources of data in the literature are heterogeneous, meta-analysis may not be used.

Contact details for further information

Xiyu Feng
u6453474@anu.edu.au

Organisational affiliation of the review

Research School of Population Health, Australian National University
http://rsph.anu.edu.au

Review team members and their organisational affiliations

Miss Xiyu Feng. Research School of Population Health; Australian National University
Dr Matthew Kelly. Research School of Population Health; Australian National University
Dr Haribondhu Sarma. Research School of Population Health; Australian National University

Type and method of review

Systematic review

Anticipated or actual start date

07 June 2021

Anticipated completion date

06 September 2021

Funding sources/sponsors

No funding.

Conflicts of interest

Language

English

Country

Australia

Stage of review

Review Ongoing

Subject index terms status

Subject indexing assigned by CRD

Subject index terms

MeSH headings have not been applied to this record

Date of registration in PROSPERO

08 July 2021

Date of first submission

08 June 2021

Stage of review at time of this submission

Stage	Started	Completed
Preliminary searches	Yes	No
Piloting of the study selection process	Yes	No
Formal screening of search results against eligibility criteria	Yes	No
Data extraction	No	No
Risk of bias (quality) assessment	No	No
Data analysis	No	No

The record owner confirms that the information they have supplied for this submission is accurate and complete and they understand that deliberate provision of inaccurate information or omission of data may be construed as scientific misconduct.

The record owner confirms that they will update the status of the review when it is completed and will add publication details in due course.

Versions

08 July 2021
08 July 2021
