# Supplementary material for: The association between educational level and multimorbidity among adults in Southeast Asia: A systematic review
Source: PLoS One. 2021 Dec 20;16(12):e0261584. doi: 10.1371/journal.pone.0261584 (PMC8687566; doi:10.1371/journal.pone.0261584)
Supplement: Attachment — Submitted filename: Response to reviewers.docx [file pone.0261584.s007.docx]

Dear reviewers

Thank you for your comments and suggestions to improve our paper. As requested by the reviewers we have thoroughly edited the whole paper with an overall aim to improve the conciseness and readability. We address the specific comments below. Our responses are in blue font, and new texts in the paper are in red font.

-Introduction:

1. Line 69 and 71: References for higher mortality rate and not receiving cost-effective treatment were missing

Thanks for pointing that out we have now added the reference as follows:

Line 73-77: “Due to the complexity of symptoms and higher mortality rates, the treatment requirements for multimorbidity are more complicated than those for single diseases, and patients with multimorbidity often do not receive cost-effective treatment [5].”

2. Line 69, line 78: The sentence is way too long for readers to follow

We have rewritten those sentences as follows:

Line 73-77: “Due to the complexity of symptoms and higher mortality rates, the treatment requirements for multimorbidity are more complicated than those for single diseases, and patients with multimorbidity often do not receive cost-effective treatment [5]. This situation may increase the economic and medical burden of these patients and lead to a reduced quality of life and greater damage to their physical and mental health [5, 6].”

Line 86-90: “Socio-economic development is connected to epidemiological transition and the growth of the prevalence of chronic diseases [9]. Moreover, inequalities in socioeconomic status (SES) may be reflected in unequal access to health care, participation in health activities, and life stressors, which would contribute to an increased burden of multiple chronic conditions [9, 10].”

-Method:

1. Please revise the entire article, especially the method session, in a concise fashion.

We have tried to make the Method section more concise. However, due to the complicated definitions and relationships we were measuring, we still need to retain some necessary detail. Please see the changed text from Line 127 to 302 in the file of “Revised Manuscript with Track Changes”.

2. Line 174 to 204: the details could be put into a table or list.

Thank you for that suggestion, please see the new tables and explanatory text below.

Line 210 to 219: “The examined selection of observational studies according to NOS was shown in the table below (Table 1) [19, 21]. Observational studies included cross-sectional studies, cohort studies and case-control studies. The selection table was based on a summary of the scored items for each of the observational studies in NOS (Additional file 2), with reference to other systematic reviews of multimorbidity that have applied NOS as an assessment tool to evaluate and examine the literature [19 21].”

**Table 1 The examined selection of observational studies according to NOS.**

|  | **Cross-sectional studies**  (Seven items,  maximum ten points) | **Cohort studies**  (Eight items,  maximum nine points) | **Case-control studies**  (Eight items,  maximum nine points) |
| --- | --- | --- | --- |
|  | | | |
| **Selection** | (Up to five points) | (Up to four points) | (Up to four points) |
|  | Representativeness; | Representativeness; | adequate definition; |
|  | Sample size; | Non exposed cohort; | Representativeness; |
|  | Non-respondents; | Ascertainment; | Selection of controls; |
|  | Ascertainment. | Demonstration. | Definition of controls. |
|  | | | |
| **Comparability** | (Up to two points) | (Up to two points) | (Up to two points) |
|  | On the basis of the study design or analysis and the control of confounders. | On the basis of the design or analysis. | On the basis of the design or analysis. |
|  | | | |
| **Outcome/Exposure** | (Up to three points) | (Up to three points) | (Up to three points) |
|  | Assessment; | Assessment; | Ascertainment; |
|  | Statistical test. | Enough long follow-up; | Same method of ascertainment; |
|  |  | Adequacy. | Non-response rate. |

Line 224 to 229: “There were three levels of quality to assess the scores of the individual study in the table below (Table 2) [19, 21]. The scores of NOS corresponding to the three levels mentioned in the table were summarized from similar levels and scores that appeared in other systematic reviews of multimorbidity [19, 21].”

**Table 2 Three levels of quality to assess the scores of the selected studies.**

|  | **Selection** | **Comparability** | **Outcome/Exposure** | **Total** |
| --- | --- | --- | --- | --- |
| **Good** | Four points or above | One point or above | Two points or above | Seven points and above |
| **Fair** | Two to three points | One point | One to two points | Five to six points |
| **Poor** | Zero to one point | Zero point | Zero point | Zero to four points |

3. Line 221 to 235, Line 247 to 251: Please rewrite it concisely.

We have now shortened this section. Please see the changed text from Line 252 to 302 and from 690 to 694 in the file of “Revised Manuscript with Track Changes”.

Line 252 to 263: “The findings regarding methods, exposures, and outcomes were described using narrative synthesis [18, 22]. The prevalence of multimorbidity was extracted from individual studies. The extraction of the prevalence of multimorbidity was divided into three cases in these studies. (1) The prevalence of multimorbidity was reported directly in the study, thus we could extract data on the prevalence of multimorbidity directly from the article. (2) If the prevalence of multimorbidity was not directly reported in the study but it reported the total number of participants and the number of people with multimorbidity, we calculated this indicator using the number of participants as the denominator and the number of people with multimorbidity as the numerator. (3) If studies did not report the prevalence of multimorbidity, the total number of participants and the number of multimorbidity cases, we categorised those studies “not available” (N/A).”

Line 286 to 294: “The available data regarding education level in the survey area were pooled in the same way to extract and calculate the prevalence (%) of multimorbidity at different education attainments. The prevalence of multimorbidity (%) at different education levels in the study was all obtained by calculation in selected studies. The prevalence of multimorbidity for a particular education level group was calculated by dividing the number of participants experiencing multimorbidity at that educational attainment level by the total sample number of participants. For studies that we could not calculate the prevalence of multimorbidity (%) at different educational levels because of lack of reporting the number of patients with multimorbidity (numerator) at different educational levels, we would put "N/A" to show the prevalence.”

Line 690 to 694: “**^*^** If the type of studies could not extract or calculate the prevalence of multimorbidity, the cumulative incidence of multimorbidity (CI, %) could be extracted directly from the study or calculated. The formula was calculated by dividing the number of new multimorbidity cases in a given period by the number of subjects at risk in the population initially at risk of multimorbidity at the start of the study. If the CI for multimorbidity could neither be extracted nor calculated from the study, it should be denoted by N/A.”

“**^#^** If the type of studies could not extract or calculate the prevalence (%) of multimorbidity at different educational levels, it was treated in the same way as if the type of studies could not extract or calculate the prevalence of multimorbidity.”

-Results:

1. The resolution of graph 1 could be improved

It has been improved. Please see the Fig 1 PRISMA flow diagram.

2. Please do not repeat all the information listed in the table; presenting the most critical findings could make this article clearer.

We have also now edited the Results to attempt to avoid repetition of the table contents. Please see the changed text from Line 316 to 439 in the file of “Revised Manuscript with Track Changes”.

-Discussion

1. Line 396: The logic development of this argument is unclear. How does the different grouping method eliminate the potential association?

We apologise our meaning here was not clear. To clarify this point, some changes have been made to the original text, which were shown below.

Line 446-451: “We identified a small number of relevant publications and found heterogeneity between these studies. For example, the estimation of sample size, the grouping of education levels, the age groups of study participants, and the inclusion and exclusion criteria varied considerably among the studies we included, making comparability difficult [18] that did not allow us to clarify the association between education level and multimorbidity using meta-analysis.”

Line 454-457: “The reasons for these results may be related to differences in the purpose and methods of these studies, such as different patterns of multimorbidity, classification of education levels, and the confounding factors, and may also be related to the databases used in these studies.”

Line 470-478: “That may be because of different groupings of educational levels and different definitions of multimorbidity. On the other hand, the patterns of multimorbidity and confounders differed between studies, as did the study outcomes [23, 27]. Even when a common data source was used for the analysis, the definition of multimorbidity, adjustment factors, and differences in education level, may impact on the results [27, 34-36]. Furthermore, the few studies from Singapore used only two databases, the Singapore Mental Health Study (SMHS) and Well-being of the Singapore Elderly (WiSE), thus it may also be possible that the association between education level and multimorbidity was not evident in these two databases [23, 27, 34-36].”

2. Line 411: How do the different times of data collection impact the association? Please elaborate more on this.

We apologize that it was not clear what we meant here. To clarify this, we have made some changes to the original text, which were shown below.

Line 486-498: “While all four Indonesia studies found [24, 28, 30, 31] that high education levels were associated with developing multimorbidity, all of them used the Indonesian Family Life Survey (IFLS). It was likely that the association between higher education level and higher odds of developing multimorbidity was evident in the IFLS database. However, there were still many differences between the results of these studies. The reasons for this include factors such as the purpose of the study, the selection of the population, and the method of analysis affected the association between multimorbidity and education level. Also relevant was that the ILFS is a longitudinal study collecting data every 4-5 years. The various studies using ILFS data conducted cross sectional analysis on various waves of this study, which may also help explain the variation. A typical example was the study by Marthias *et al* [31]. This study consisted of two cross-sectional sub-studies whose purpose and methods were consistent. However, the difference in the pattern of multimorbidity and participants between these two sub-studies made the results of the association between multimorbidity and education level different [31].”

3. Line 426: The reference you cited here is a meta-analysis on 24 cross-sectional studies; this study design is unlikely to provide proof for causal correlation. Please check.

In this article, the reference [8], a meta-analysis of 24 cross-sectional studies, was used with other relevant sources [9, 10] to show the association of socioeconomic inequality and educational inequality on multimorbidity (Line 80-92). In addition, this reference was also used separately to show that “high levels of education have been reported to reduce the odds of developing multimorbidity in western countries such as the United States and Canada (Line 546).”, as this was one of the reference’s results. Since this reference was a meta-analysis of 24 cross-sectional studies and was unlikely to reflect proof of causality, we apologize for our inaccurate presentation here. To clarify this point, we have made some changes to this, which were shown below.

Line 510-5`3: “The improvement of educational level was recognized as reducing risk of multimorbidity [10, 44]. The potential reason would be that as the level of education increased, people's SES would also increase, making them more aware of healthy living and gaining health literacy and decreasing the risk [8, 10, 44].”
