# Supplementary material for: The association between educational level and multimorbidity among adults in Southeast Asia: A systematic review
Source: PLoS One. 2021 Dec 20;16(12):e0261584. doi: 10.1371/journal.pone.0261584 (PMC8687566; doi:10.1371/journal.pone.0261584)
Supplement: Attachment — Submitted filename: Response to Reviewers.docx [file pone.0261584.s009.docx]

Dear Reviewers

Thank you so much for your comments and suggestions on our paper. As requested by the reviewers, we have further edited the entire paper to generally improve its readability. We have discussed specific comments below. Our responses are shown in blue font, and new text in the paper is shown in red font.

-Methods

Line 130: The authors describe in results section that they have found studies with individuals over 18 years old; in this case, why the criterion of 15 years old or more? Any studies with individuals under 18 years old?

Thank you very much for your comments, there are two studies where the age of participants was specified as over 15 years rather than over 18 years, namely the study by Ba *et al* [26] and the study by Tiptaradol and Aekplakorn [37]. We apologise our description here may not be appropriate. We have reviewed this sentence.

Line 130-131: Inclusion criteria: “(6) participants in studies being over 15 years of age.”

Line 142: Exclusion criteria: “(8) participants in studies was younger than 15 years.”

Line 173-174: Remember: work already was done, so, the verb tense must be put in the past.

Thanks so much for your comments. We apologize for the error in our verb tense here. And thank you very much for pointing out this error.

-Results

Line 247 to 249: I believe this should be an exclusion criterion.

Thank you very much for your comment. It has been added to the exclusion criteria. Accordingly, we have also made a corresponding change to the inclusion criteria, as the exclusion criteria are the opposite to the inclusion criteria presented.

Line 122 to 123: Inclusion criteria: “(3) studies reporting or having available detailed data on associations between level of education and multimorbidity.”

Line 136 to 138: Exclusion criteria: “(5) studies where detailed data was not available regarding the association between educational level and multimorbidity.”

Line 286: Any study under 18 years old?

Thank you very much for your comments. In the study by Ba *et al* [26] and in the study by Tiptaradol and Aekplakorn [37], the participants were older than 15 years, not older than 18 years. Since only two of the 18 studies included in this paper had an age of 15 years or older, we have focused here only on participants older than 18 years. We apologise our meaning here was not clear and have revised this.

Line 286-287: “The prevalence of multimorbidity ranged from 3.2% to 72.6% among over 15 years-old participants.”

-Discussion

Line 351: I suggest describing briefly these two obvious associations, in order to refresh readers about the main results have been found.

Thank you very much for your comment. It has been added.

Line 350 to 353: “There were two obvious associations between education and multimorbidity in Singapore and Indonesia, respectively. Firstly, educational level was not associated with multimorbidity in Singapore, alternately higher levels of education were associated with higher odds of developing multimorbidity in Indonesia.”

Line 357-359: Did the authors not mention in their discussion the possible causes of non-association?

Thank you very much for your comment, our discussion in this area was not accurate enough. We have made additions and corrections.

Line 367-383: “Most studies in Singapore [23, 35, 36] mentioned that education level was not associated with multimorbidity. According to the authors, this is likely to be related to the fact that these studies [23, 27, 34-36] from Singapore used only two databases, the Singapore Mental Health Study (SMHS) [23, 27, 35] and the Wellbeing of the Elderly in Singapore (WiSE) [34, 36]. It may be that the association between education level and multimorbidity was not significant in these two databases [23, 27, 34-36]. In addition, it is worth noting that the results would be influenced by many factors, even when a common data source was used for the analysis, the definition of multimorbidity, adjustment factors, and differences in education level, may impact on the results. For example, the study of Chong *et al* [27] and the study of Subramaniam *et al* [35] used the SMHS-2010, but the classification of education level (the study of Subramaniam *et al* [35] combining the level of Pre-U/Junior College/Diploma together) and the pattern of multimorbidity differed (the pattern of multimorbidity including mental disorders in the study of Chong *et al* [27]), thus the results of the connection of multimorbidity and education of these two studies were different. Similarly, the study of Picco *et al* [34] and the study of Subramaniam *et al* [36] used WiSE, but their patterns of multimorbidity and adjustment factors were very different, leading to distinct results of the association between educational level and multimorbidity.”

Line 459-460: What should be noted?

Thank you very much for your comment. The note here refers to the fact that self-reporting having recall bias may have an impact on the results of the association between multimorbidity and education. We apologize that this statement here may have been misleading to readers, so we have deleted it.

Line 466-468: Are the authors sure about that? What the true picture?

Thank you very much for your comment. We apologize that the use of "true picture" here may be misleading to readers. We wanted to convey that the larger the sample size, the closer to accurate the results should be, controlling for other variables such as age and data collection. We have made the change here.

Line 468 to 470: “However, in general, the larger the sample size, the more accurate the results should be, controlling for other variables such as age and data collection [8, 19, 22].”

Line 519: I believe the authors did not assess the methodological adequacy of the studies. It was not the scope of the study. In this case, I would be more cautious and would only indicate that the heterogeneity of the findings did not allow to reach a definitive conclusion about the association between educational level and multimorbidity.

Thanks so much for your comments. Your comment makes a lot of sense, to which we have made some changes here.

Line 517 to 522: “This study is a comprehensive mapping of research related to the association between education and multimorbidity in the Southeast Asian region and reveals the neglect and lack of studies on the connection between multimorbidity and educational level in this region. The heterogeneity of the findings did not allow us to reach a definitive conclusion about the association between educational level and multimorbidity.”
